# Dendrite glia interactions: lessons from the *C. elegans* amphid sense organ

**DOI:** 10.3389/fcell.2025.1715817

**Published:** 2025-11-20

**Authors:** Katherine C. Varandas

**Affiliations:** Department of Biological Sciences, Seton Hall University, South Orange, NJ, United States

**Keywords:** dendrite, glia, *C. elegans*, sense organ, amphid

## Abstract

Glia are critical components of the nervous system, regulating the development and function of associated neurons. While much attention has focused on interactions between glia and axons, growing evidence highlights the importance of critical and evolutionarily conserved interactions between glia and dendrites, particularly in organisms with simple nervous systems such as *Caenorhabditis elegans*. Glia critically support the structure and function of associated dendrites through regulation of the ionic microenvironment, uptake of extracellular vesicles and fragments, and signaling regulation downstream of direct glial sensation of environmental stimuli in the major *C. elegans* sense organs. Glia also elicit beneficial responses upon defects in dendrite structure, stress, aging, and perhaps exposure to pathogens. Emerging themes are that a single glial cell can regulate distinct interacting dendrites differently and that neurons can communicate extra-synaptically via a shared interacting glial cell.

## 1 Introduction

The remarkable functions of the nervous system rely on polarization of neurons into specialized domains for signal reception, dendrites, and signal transmission, axons. Axons and dendrites have distinct protein repertoires and structures to support their functions. Neurons interact extensively with glia, which are defined by three criteria: (1) physical association with neurons, (2) lack of signaling via fast currents or release of neurotransmitter-filled vesicles, and (3) shared developmental precursor cells with neurons, with the exception of microglia (Shaham, 2006; Mehl et al., 2022). While attention has focused on interactions between glia and axons (Hertzler and Rolls, 2024), animals with simple nervous systems, such as *Caenorhabditis elegans,* suggest important and conserved interactions between glia and dendrites (Heiman and Shaham, 2007). Changes in dendrite structure correlate with learning and memory (Bernardinelli et al., 2014) and dendrite defects occur in many nervous system diseases (Kulkarni and Firestein, 2012). Therefore, understanding how dendrite structure and function may be influenced by interacting glia is critical.

The experimental advantages of *C. elegans* make it a powerful experimental system (Brenner, 1974), with specific advantages for studying dendrite glia interactions. In addition to a fully anatomically and molecularly mapped nervous system (Ward et al., 1975; White et al., 1986; Hall and Russell, 1991; Hammarlund et al., 2018; Cook et al., 2019; Taylor et al., 2021; Purice et al., 2025), *C. elegans* is genetically tractable and optically transparent, enabling gene discovery and high-resolution imaging of intact organisms, respectively. Despite its simple nervous system, *C. elegans* exhibits complex behaviors including sensory discrimination, locomotion, sleep, mating, decision making, and memory (Bargmann, 2006; Raizen et al., 2008; Ardiel and Rankin, 2010; Emmons, 2018; Haspel et al., 2020). Notably, *C. elegans* glia do not provide trophic support to neurons (Shaham, 2005; Barres, 2008), allowing for discovery of non-trophic glial functions via ablation or other manipulations (Singhvi and Shaham, 2019). Unlike *C. elegans* axons which largely regenerate after breakage, ciliated dendrites show little capacity for regrowth (Chung et al., 2006; 2016), suggesting dendrite glia interactions may be critical to maintain dendrite integrity.

Every *C. elegans* glial cell is part of a sense organ. *Caenorhabditis elegans* sense organs share morphological and functional similarities to mammalian epithelial sense organs such as the olfactory epithelia, retina, cochlea, and taste buds (Heiman and Bülow, 2024). They are composed of sensory neurons with ciliated dendrites, which detect environmental cues including tastes, smells, temperature, pheromones, oxygen, and some aspects of touch (Bargmann, 2006; Goodman et al., 2019; Ferkey et al., 2021), and associated glia (Ward et al., 1975). Different sensory dendrite cilia contain distinct receptors and downstream signaling proteins, allowing for diverse sensory capabilities, and many are morphologically elaborated (Goodman and Sengupta, 2019; Ferkey et al., 2021; Maurya, 2022).

**FIGURE 1:**
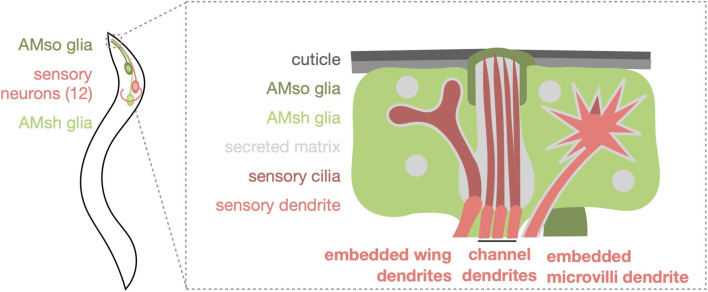
Schematic representation of the *Caenorhabditis elegans* amphid sense organ. One of the two bilaterally symmetric amphids is shown for simplicity. Three amphid sensory neurons have embedded wing dendrites (AWA, AWB, and AWC). Eight amphid sensory neurons have channel dendrites, six of which have a singlet cilium (ASE, ASG, ASH, ASI, ASJ, and ASK) and two have doublet cilia (ADF and ADL). The AFD neuron has an embedded, actin-rich microvilli dendrite and simple cilium. All dendrite endings except for AFD are directly exposed to the environment by the channel formed by AMso glia at the tip and AMsh glia at the base.

The largest and best-characterized sense organs of *C. elegans* are the bilaterally symmetric amphids. Each amphid contains twelve sensory neurons and two glia (Figure 1). Amphid sensory neurons are bipolar, with an axon projecting into the brain-like neuropil and a ciliated dendrite projecting towards the animal surface. The dendrite endings (DEs) of amphid neurons fall into two classes: 8 possess channel dendrites, whose simple singlet or doublet cilia extend through a glia-formed channel for direct environment exposure, and the remaining 4 are embedded in hand-in-glove configurations within the AMsh glia (Oikonomou and Shaham, 2011). Three of the embedded DEs, those of AWA, AWB, and AWC, are elaborated cilia exposed the environment, while the DE of AFD contains thermosensory actin-rich microvilli and a simple cilium not exposed to the environment (Ward et al., 1975; Doroquez et al., 2014). The amphid sheath (AMsh) glia form the base of the tube-shaped sensory channel and are highly secretory (Wallace et al., 2016). The amphid socket (AMso) glia form the distal tip of the tube, sculpting an open channel that connects with the external cuticle (Singhvi et al., 2024).

This minireview focuses on the bidirectional communication between dendrites and glia in the healthy amphid and upon dendrite structure defects, stress, aging, and pathogen exposure, focusing interactions outside of organism development.

## 2 Dendrite glia interactions in the healthy amphid

### 2.1 Glia support distinct interacting dendrites differently

**FIGURE 2:**
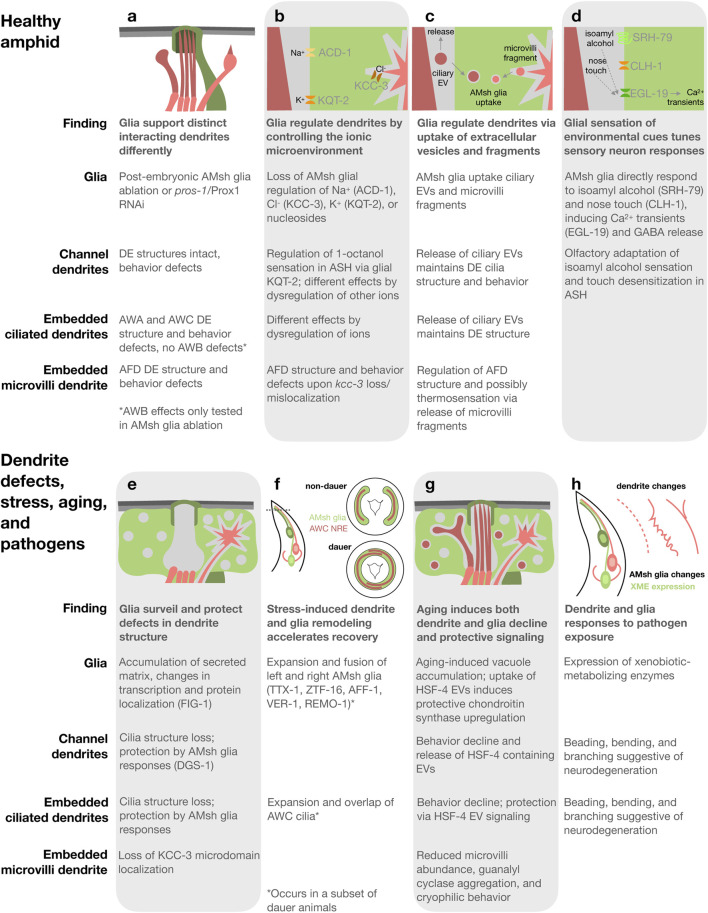
Summary and schematics of dendrite glia interactions in the healthy *C. elegans* amphid sense organs **(a–d)** and upon defects in dendrite structure **(e)**, stress-induced dauer **(f)**, aging **(g)**, and pathogen exposure **(h)**. Proteins involved in each process in parentheses.

In the amphid, glia support the structure and function of distinct interacting dendrites differently (Figure 2a). Ablation of AMsh glia after amphid formation (in first-stage larvae) causes structural defects in a subset of the embedded DEs. The elaborated cilia of AWA and AWC as well as the actin-rich microvilli of AFD show loss or reduction in structural elaboration, while the elaborated cilia of AWB remain intact. Ablation-induced dendrite structural changes correlate with functional deficits; chemosensory and thermosensory behaviors mediated by the structurally impacted DEs are disrupted or altered, while behaviors mediated by AWB remain intact. In contrast, although the structure of channel neuron DEs and the localization of signaling proteins to them remains largely unaffected, behaviors mediated by these neurons are nevertheless impaired (Bacaj et al., 2008). Similar effects are observed in animals upon loss of *pros-1*/Prox1, a conserved homeodomain transcription factor enriched in AMsh glia. PROS-1 drives expression of many secreted and transmembrane proteins critical for amphid function. Animals treated post-embryonically with *pros-1* RNAi exhibit defects in the structure of embedded DEs and their associated behaviors, while channel neuron structures remain preserved yet associated behaviors are disrupted. *pros-1* RNAi causes severe ultrastructural abnormalities in the amphid channel, which likely underlie the similarity in effects to AMsh glia ablation (Wallace et al., 2016). Both AMsh glia-ablation and *pros-1* loss illustrate that glia support the structure and function of distinct interacting dendrites differently and suggest a critical role for glia secreted and transmembrane proteins in dendrite support.

### 2.2 Glia regulate dendrites by controlling the ionic microenvironment

Glia regulate the local concentration of extracellular ions, or ionic microenvironment, of both synapses and sites of sensory input, which impacts neuron development and activity as well as circuit dynamics (Ray and Singhvi, 2021). Indeed, several ion channels and transporters function in AMsh glia to regulate dendrite structure and function in the amphid (Figure 2b). A recent systematic study revealed that AMsh glial ion channels and transporters, specifically those regulating K^+^, Cl^−^, and nucleosides, affect distinct neurons differently (Wang et al., 2022). Knockout of *acd-1*, a proton-gated Na^+^ channel subunit of the DEG/ENaC family expressed in AMsh glia, exacerbates sensory deficits caused by mutations in neuronal sensory signaling machinery, indicating that glial ACD-1 supports neuron activity (Wang et al., 2008; Wang et al., 2012). The K^+^/Cl^−^ co-transporter KCC-3/SLC124A4 is required in AMsh glia for the structural elaboration of AFD microvilli. KCC-3 regulates local Cl^−^ concentration to control AFD microvilli shape (Singhvi et al., 2016). AFD is the primary thermosensory neuron in *C. elegans* and *kcc-3* mutants have defects in thermotaxis, indicating that KCC-3 is critical for AFD function (Singhvi et al., 2016; Yoshida et al., 2016). The voltage-gated K^+^ (KCNQ) channel KQT-2 controls the resting membrane potential of AMsh glia, which regulates AMsh glia Ca^2+^ influx via EGL-19 and subsequent GABA release. This consequently controls response of the channel dendrite neuron ASH to the aversive odorant 1-octanol. Expression of human KCNQ channels in AMsh glia rescues *kqt-2* mutation, indicating conservation. Additionally, expressing KCNQ with mutations from patients with developmental and epileptic encephalopathy causes glial, neuronal, and behavioral phenotypes and treatment of these animals with a KCNQ channel opening drug exerts rescuing effects on 1-octanol sensation (Graziano et al., 2024). These studies demonstrate that glia impact distinct associated dendrites differently via their control of the ionic microenvironment and underscore the value of the *C. elegans* amphid as a model for investigating conserved dendrite glia interaction mechanisms.

### 2.3 Glia form microdomains surrounding distinct dendrite endings

AMsh glia create unique microdomains surrounding distinct dendrite endings. The K^+^/Cl^−^ co-transporter KCC-3/SLC124A4 localizes specifically to AMsh glia membranes surrounding the actin-rich microvilli of AFD dendrites (Figure 2b; Singhvi et al., 2016). Localization to this microdomain is regulated not by AFD neurons nor their microvilli, but by restriction to this microdomain by the dendrite cilia of non-AFD dendrites (Ray et al., 2024). A microdomain surrounding the channel DEs is enriched in the secreted protein VAP-1, the transmembrane proteins DAF-6/PATHD3 and CHE-14/Dispatched, and actin cortex-associated kinase LIT-1/NLK (Perens and Shaham, 2005; Oikonomou et al., 2011; Ray et al., 2024). The microdomain surrounding the embedded wing DE of AWC is defined as enriched in neither VAP-1 nor KCC-3/SLC124A4. Regulation of AMsh glia microdomains by the dendrites of other microdomains indicates that neurons communicate extra-synaptically through a shared glial cell (Ray et al., 2024). Determining if there are unique protein repertoires surrounding the embedded wing DEs that regulate their elaborated structures and whether ion channels and transporters other than KCC-3/SLC124A4 exhibit microdomain localization are interesting avenues for future study.

### 2.4 Glia regulate dendrites via uptake of extracellular vesicles and fragments

Extracellular vesicles (EVs) are released by diverse cell types, facilitating cellular sculpting and intercellular communication. EVs are released from the cilia of many, if not all, *C. elegans* sensory neurons (Wang et al., 2024). In the amphid, both channel and embedded dendrite cilia produce EVs that are subsequently released into the environment or engulfed by AMsh glia (Figure 2c; Ohkura and Bürglin, 2011; Razzauti and Laurent, 2021). These EVs are marked by tetraspanins homologous to mammalian EV markers, TSP-6/CD9 and TSP-7/CD63. EV uptake by AMsh glia requires ATP, indicating an active membrane trafficking process. Interestingly, mutants lacking proper cilia structures accumulate more AMsh glia-engulfed EVs than wild type. Inhibition of AMsh glia endocytosis causes misshapen DE cilia and disruption of channel, but not embedded, dendrite neuron-associated behaviors. Therefore, glial uptake of ciliary EVs maintains the structure and function of associated dendrite cilia (Razzauti and Laurent, 2021).

The actin-rich microvilli of embedded AFD dendrites also shed fragments taken up by AMsh glia (Figure 2c; Raiders et al., 2021; Razzauti and Laurent, 2021). TSP-6/CD9 and TSP-7/CD63 also label these fragments (Razzauti and Laurent, 2021), indicating that they may arise via a process shared with ciliary EVs. Raiders et al. found that glia engulf fewer fragments from active AFD neurons than silenced neurons, resulting in longer microvilli and altered thermosensation. Phosphatidyl serine exposure on the AFD membrane outer leaflet signals engulfment to AMsh glia, which utilize apoptotic engulfment proteins including CED-10/Rac1 (Raiders et al., 2021). Razzuati and Laurent found that upon AMsh glia ablation, AFD microvilli continue to shed fragments taken up instead by other surrounding cells and that blocking AMsh glial endocytosis causes misshapen AFD endings, but does not affect thermotaxis behavior. These studies provide conflicting data on the necessity of glial uptake of microvilli-derived fragments for thermosensation. In contrast to cilia mutants, mutants defective in AFD microvilli structure accumulate fewer AMsh glia-engulfed neuronal fragments (Razzauti and Laurent, 2021). The sculpting of amphid DEs by glia, both cilia and microvilli, is reminiscent of glial synaptic sculpting in *Drosophila* and vertebrates (Shaham, 2010; Wilton et al., 2019; Hilu-Dadia and Kurant, 2020; Singhvi et al., 2024).

### 2.5 Glial sensation of environmental cues tunes interacting sensory neuron responses

AMsh glia directly detect environmental cues. AMsh glia sense aversive odorants (Duan et al., 2020) and nose touch (Ding et al., 2015; Fernandez-Abascal et al., 2022) and subsequently tune the responses of associated sensory neurons (Figure 2d), in addition to responding to stress (Procko et al., 2011) and pathogens (Wallace et al., 2021), as discussed later. AMsh glia directly respond to aversive concentrations of the odorant isoamyl alcohol via SRH-79, a receptor distinct from that in sensory neurons, leading to Ca^2+^ transients. This results in GABA release from AMsh glia, which inhibits the associated ASH neuron thereby suppressing aversive-odorant triggered avoidance and promoting olfactory adaptation (Duan et al., 2020). Similarly, in response to nose touch, AMsh glia exhibit Ca^2+^ transients that require the Cl^−^ channel CLH-1/ClC-2 (Fernandez-Abascal et al., 2022), but not Na^+^ channels (Ding et al., 2015) or PEZO-1/PIEZO1/2 (Fernandez-Abascal et al., 2025). Cl- ion efflux from AMsh glia via CLH-1/ClC-2 in response to nose touch is required for glial GABA-mediated inhibition and regulation of cyclic AMP in associated touch-sensitive ASH neurons and subsequent touch desensitization (Fernandez-Abascal et al., 2022). Both olfactory and tactile stimuli-evoked Ca2+ transients in AMsh glia require the voltage-gated calcium channel EGL-19, homologous to the vertebrate ɑ1 subunit of L-type voltage-gated calcium channels (Chen et al., 2022). In summary, direct glial sensation of environmental stimuli regulates associated neuron activity and downstream responses in the *C. elegans* amphid, as has been previously demonstrated for glia and glia-like accessory cells of vertebrate touch receptors and *Drosophila* olfactory sensilla (Ackerman et al., 2022).

## 3 Dendrite glia interactions upon dendrite defects, stress, aging, and pathogen exposure

### 3.1 Glia surveil and protect defects in dendrite structure

Glia detect and respond protectively to defects in the structure of associated dendrites in the amphid. Electron microscopy revealed that an electron-dense matrix accumulates in and around the AMsh glia in animals with sensory dendrite cilia mutations, which cause defects in cilia structure and impaired sensory function (Lewis and Hodgkin, 1977; Perkins et al., 1986). Indeed, glia acutely accumulate secreted matrix and alter their transcription when ensheathing dendrites with cilia defects (Figure 2e; Varandas et al., 2025). The localization of several AMsh glial proteins is also disrupted in cilia mutants, including the channel microdomain proteins DAF-6/PATHD3 and LIT-1/NLK (Perens and Shaham, 2005; Oikonomou et al., 2011) and the AFD microdomain protein KCC-3 (Ray et al., 2024), indicating additional responses. A previously uncharacterized 7-transmembrane domain protein found on a subset of channel dendrite cilia, DGS-1, signals the presence of intact dendrite cilia to FIG-1, a thrombospondin domain-containing transmembrane protein found on AMsh glia membranes surrounding channel DEs (Varandas et al., 2025). Intriguingly, DGS-1 and FIG-1 closely resemble the distinct peptides formed by autoproteolysis of the brain-specific angiogenesis inhibitors, a subfamily of vertebrate adhesion G protein-coupled receptors (Stephenson et al., 2014; Varandas et al., 2025). The glial responses to dendrite structure defects protect dendrites, as the pre-existence of these responses delays acute cilia disruption (Varandas et al., 2025). Exploring whether glia detect dendrite defects and protect against their perturbation in additional contexts, such at synapses and upon neurodegeneration, is a fascinating area for future studies.

### 3.2 Stress-induced dendrite and glia remodeling accelerates recovery

The amphid undergoes remodeling of both dendrites and glia in dauer, a developmentally arrested protective stage entered in response to stresses including starvation, crowding, and high temperature (Cassada and Russell, 1975; Golden and Riddle, 1984). In a subset of dauer animals, the left and right AMsh glia expand at the nose tip and fuse, allowing cytoplasmic exchange, and the embedded DEs of AWC expand within the fused AMsh glia until the left and right overlap (Figure 2f; Albert and Riddle, 1983; Procko et al., 2011). Interestingly, AMsh glia remodeling is independent of AWC dendrite remodeling, yet AWC dendrite remodeling requires AMsh glia and their remodeling (Bacaj et al., 2008; Procko et al., 2011). AMsh glia remodeling requires the fusogen AFF-1, the transcription factors TTX-1/Otx and ZTF-16/Ikaros, the receptor tyrosine kinase VER-1/RTK, and the Srz type 7-transmembrane domain protein REMO-1 (Procko et al., 2011; Procko et al., 2012; Lee et al., 2021). Interestingly, upon exposure to favorable conditions, amphid remodeling accelerates dauer recovery (Lee et al., 2021). How amphid remodeling accelerates dauer recovery and whether similar stress-induced beneficial remodeling occurs in other settings are interesting future questions.

### 3.3 Aging induces both dendrite and glia decline and protective signaling

Cognition and behavior deteriorate upon aging. Indeed, amphid-mediated sensory behaviors deteriorate, accompanied by changes in sensory neurons and glia in aged *C. elegans* (Figure 2g). AFD microvilli reduce in abundance and thermosensory guanylyl cyclases aggregate, accompanied by cryophilic behavior (Huang et al., 2020). Amphid neuron-mediated behaviors decline, accompanied by cilia alterations and intraflagellar transport slowing (Cornils et al., 2016; Zhang et al., 2021; Wu et al., 2025). AMsh glia accumulate vacuole structures marked with late endosome and lysosomal markers, correlating with decline in embedded neuron function (Wu et al., 2025).

Protective dendrite-glia signaling via EVs during aging was recently discovered in the amphid (Figure 2g). Sensation by both channel and embedded dendrite neurons declines in aging and, interestingly, animals with early decline in channel dendrite neuron-mediated sensation have improved embedded dendrite neuron function. Aging of neurons with channel dendrites induces the release of EVs containing the heat shock protein HSP-4/BiP, which are taken up by surrounding AMsh glia. HSP-4/BiP is part of the endoplasmic reticulum unfolded protein response and when taken up by AMsh glia, activates the IRE1-XBP1 transcriptional pathway increasing the expression of chondroitin synthesis proteins. The result is protection of both AMsh glia and embedded dendrite neuron functional decline (Wu et al., 2025). Interestingly, exogenous activation of both the endoplasmic reticulum and cytosolic unfolded protein responses in another *C. elegans* glia cell type extends lifespan (Frakes et al., 2020; Gildea et al., 2022), highlighting the importance of glial protein quality for healthy aging. Exploring whether dendrite to glia signaling via EVs is employed upon aging and whether exogenous activation of the glial unfolded protein response, perhaps via EVs, elicits protective effects in other settings are exciting questions for future study.

### 3.4 Dendrite and glia responses to pathogen exposure

Sense organs connect the nervous system to the environment and are therefore sites where neurons and glia are directly exposed to microbes including pathogens. Upon exposure to the pathogenic bacteria *Pseudomonas aeruginosa*, sensory function of ASE neurons becomes impaired and both embedded and channel dendrites exhibit beading, bending, and branching suggestive of neurodegeneration (Figure 2h; Wu et al., 2015). The pathogenic mold *Penicillium brevicompactum* and the bacteria *Serratia marcescens* induce transcriptional changes including expression of xenobiotic-metabolizing enzymes in AMsh glia (Figure 2h) and the intestine. Neuronal cilia are not required for the glial response to these microbes, indicating that glia directly sense them. If xenobiotic-metabolizing enzyme expression cannot be induced in the intestine, the previously tolerable *Penicillium brevicompactum* becomes toxic (Wallace et al., 2021). Future studies of how pathogen responses in neurons, glia, and distal tissues like the intestine are interrelated and how responses in sense organs impact organism health are warranted.

## 4 Concluding remarks

Studies in the *C. elegans* amphid sense organs reveal that glia critically support the structure and function of associated dendrites in the healthy amphid through regulation of the ionic microenvironment, uptake of extracellular vesicles and fragments, and activity regulation downstream of direct glial sensation of environmental stimuli. Glia also elicit beneficial responses upon defects in dendrite structure, stress, aging, and perhaps exposure to pathogens. An emerging theme is how a single glial cell, the AMsh glia, impacts distinct interacting dendrites differently. The formation of glial microdomains surrounding distinct dendrites as well as the unique protein repertoires of interacting dendrites likely contribute to glia’s different effects on these dendrites. AMsh glia also facilitate communication between neurons that do not connect via synapses, both by the control of microdomain composition and by signaling downstream of EV uptake upon aging. The mechanisms described here all occur between a single glial cell, the AMsh, and its twelve associated dendrites. Therefore, an interesting future question is whether the other amphid glial cell, AMso, also impacts associated dendrites. While the conservation of several mechanisms have been described, exploring whether similar dendrite glia interaction mechanisms occur at synapses, which have structural and molecular similarities to sense organs (Shaham, 2010), or in additional organisms present exciting areas for future study.

## References

[B1] Ackerman S. D. Singhvi A. Bianchi L. (2022). Editorial: accessory cells of sensory systems and their functional roles. Front. Neurosci. 16, 965580. 10.3389/FNINS.2022.965580 35844212 PMC9281577

[B2] Albert P. S. Riddle D. L. (1983). Developmental alterations in sensory neuroanatomy of the *Caenorhabditis elegans* dauer larva. J. Comp. Neurology 219, 461–481. 10.1002/CNE.902190407 6643716

[B3] Ardiel E. L. Rankin C. H. (2010). An elegant mind: learning and memory in *Caenorhabditis elegans* . Learn. and Mem. 17, 191–201. 10.1101/LM.960510 20335372

[B4] Bacaj T. Tevlin M. Lu Y. Shaham S. (2008). Glia are essential for sensory organ function in *C. elegans* . Science 322, 744–747. 10.1126/science.1163074 18974354 PMC2735448

[B5] Bargmann C. I. (2006). Chemosensation in *C. elegans* . WormBook: the online review of C. elegans biology. Pasadena, CA: WormBook, 1–29. 10.1895/wormbook.1.123.1 PMC4781564 18050433

[B6] Barres B. A. (2008). The mystery and magic of glia: a perspective on their roles in health and disease. Neuron 60, 430–440. 10.1016/J.NEURON.2008.10.013 18995817

[B7] Bernardinelli Y. Nikonenko I. Muller D. (2014). Structural plasticity: mechanisms and contribution to developmental psychiatric disorders. Front. Neuroanat. 8, 123. 10.3389/fnana.2014.00123 25404897 PMC4217507

[B8] Brenner S. (1974). The genetics of *Caenorhabditis elegans* . Genetics 77, 71–94. 10.1093/genetics/77.1.71 4366476 PMC1213120

[B9] Cassada R. C. Russell R. L. (1975). The dauerlarva, a post-embryonic developmental variant of the nematode *Caenorhabditis elegans* . Dev. Biol. 46, 326–342. 10.1016/0012-1606(75)90109-8 1183723

[B10] Chen D. Cheng H. Liu S. Al-Sheikh U. Fan Y. Duan D. (2022). The voltage-gated calcium channel EGL-19 acts on glia to drive olfactory adaptation. Front. Mol. Neurosci. 15, 907064. 10.3389/fnmol.2022.907064 35782381 PMC9247319

[B11] Chung S. H. Clark D. A. Gabel C. V. Mazur E. Samuel A. D. T. (2006). The role of the AFD neuron in *C. elegans* thermotaxis analyzed using femtosecond laser ablation. BMC Neurosci. 7, 30. 10.1186/1471-2202-7-30 16600041 PMC1450292

[B12] Chung S. H. Awal M. R. Shay J. McLoed M. M. Mazur E. Gabel C. V. (2016). Novel DLK-Independent neuronal regeneration in *Caenorhabditis elegans* shares links with activity-dependent ectopic outgrowth. Proc. Natl. Acad. Sci. U. S. A. 113, E2852–E2860. 10.1073/PNAS.1600564113 27078101 PMC4878464

[B13] Cook S. J. Jarrell T. A. Brittin C. A. Wang Y. Bloniarz A. E. Yakovlev M. A. (2019). Whole-animal connectomes of both *Caenorhabditis elegans* sexes. Nat. 2019 571 (7763), 63–71. 10.1038/s41586-019-1352-7 31270481 PMC6889226

[B14] Cornils A. Maurya A. K. Tereshko L. Kennedy J. Brear A. G. Prahlad V. (2016). Structural and functional recovery of sensory cilia in *C. elegans* IFT mutants upon aging. PLoS Genet. 12, e1006325. 10.1371/JOURNAL.PGEN.1006325 27906968 PMC5131903

[B15] Ding G. Zou W. Zhang H. Xue Y. Cai Y. Huang G. (2015). *In vivo* tactile stimulation-evoked responses in *Caenorhabditis elegans* amphid sheath glia. PLoS One 10, e0117114. 10.1371/JOURNAL.PONE.0117114 25671616 PMC4325002

[B16] Doroquez D. B. Berciu C. Anderson J. R. Sengupta P. Nicastro D. (2014). A high-resolution morphological and ultrastructural map of anterior sensory cilia and glia in *Caenorhabditis elegans* . Elife 2014, e01948. 10.7554/ELIFE.01948 24668170 PMC3965213

[B17] Duan D. Zhang H. Yue X. Fan Y. Xue Y. Shao J. (2020). Sensory glia detect repulsive odorants and drive olfactory adaptation. Neuron 108, 707–721. 10.1016/j.neuron.2020.08.026 32970991

[B18] Emmons S. W. (2018). Neural circuits of sexual behavior in Caenorhabditis elegans. Annu. Rev. Neurosci. 41, 349–369. 10.1146/ANNUREV-NEURO-070815-014056 29709211

[B19] Ferkey D. M. Sengupta P. L’Etoile N. D. (2021). Chemosensory signal transduction in *Caenorhabditis elegans* . Genetics 217, iyab004. 10.1093/GENETICS/IYAB004 33693646 PMC8045692

[B20] Fernandez-Abascal J. Johnson C. K. Graziano B. Wang L. Encalada N. Bianchi L. (2022). A glial ClC cl− channel mediates nose touch responses in *C. elegans* . Neuron 110, 470–485.e7. 10.1016/J.NEURON.2021.11.010 34861150 PMC8813913

[B21] Fernandez-Abascal J. Hall J. D. Bianchi L. (2025). PEZO-1 is not required for AMsh glial responses to mechanical stimulation and does not play a major role in nose touch avoidance in *C. elegans* . Micropubl. Biol. 2025. 10.17912/MICROPUB.BIOLOGY.001668 40625671 PMC12231307

[B22] Frakes A. E. Metcalf M. G. Tronnes S. U. Bar-Ziv R. Durieux J. Gildea H. K. (2020). Four glial cells regulate ER stress resistance and longevity *via* neuropeptide signaling in C. elegans. Science 367, 436–440. 10.1126/SCIENCE.AAZ6896 31974253 PMC7357615

[B23] Gildea H. K. Frankino P. A. Tronnes S. U. Pender C. L. Durieux J. Dishart J. G. (2022). Glia of *C. elegans* coordinate a protective organismal heat shock response independent of the neuronal thermosensory circuit. Sci. Adv. 8, eabq3970. 10.1126/SCIADV.ABQ3970 36490338 PMC9733925

[B24] Golden J. W. Riddle D. L. (1984). The *Caenorhabditis elegans* dauer larva: developmental effects of pheromone, food, and temperature. Dev. Biol. 102, 368–378. 10.1016/0012-1606(84)90201-X 6706004

[B25] Goodman M. B. Sengupta P. (2019). How Caenorhabditis elegans senses mechanical stress, temperature, and other physical stimuli. Genetics 212, 25–51. 10.1534/GENETICS.118.300241 31053616 PMC6499529

[B26] Graziano B. Wang L. White O. R. Kaplan D. H. Fernandez-Abascal J. Bianchi L. (2024). Glial KCNQ K+ channels control neuronal output by regulating GABA release from glia in *C. elegans* . Neuron 112, 1832–1847.e7. 10.1016/J.NEURON.2024.02.013 38460523 PMC11156561

[B27] Hall D. H. Russell R. L. (1991). The posterior nervous system of the nematode caenorhabditis elegans: serial reconstruction of identified neurons and complete pattern of synaptic interactions. J. Neurosci. 11, 1–22. 10.1523/JNEUROSCI.11-01-00001.1991 1986064 PMC6575198

[B28] Hammarlund M. Hobert O. Miller D. M. Sestan N. (2018). The CeNGEN project: the complete gene expression map of an entire nervous system. Neuron 99, 430–433. 10.1016/j.neuron.2018.07.042 30092212 PMC6576255

[B29] Hart A. C. (2006). Behavior. WormBook: the online review of C. elegans biology. Pasadena, CA: WormBook, 2005–2018. 10.1895/WORMBOOK.1.87.1

[B30] Haspel G. Deng L. Harreguy M. B. Tanvir Z. (2020). Elegantly. Neural Control Mov. Model Syst. Tools Study Locomotor Funct., 3–29. 10.1016/B978-0-12-816477-8.00001-6

[B31] Heiman M. G. Bülow H. E. (2024). Dendrite morphogenesis in *Caenorhabditis elegans* . Genetics 227, iyae056. 10.1093/GENETICS/IYAE056 38785371 PMC11151937

[B32] Heiman M. G. Shaham S. (2007). Ancestral roles of glia suggested by the nervous system of *Caenorhabditis elegans* . Neuron Glia Biol. 3, 55–61. 10.1017/S1740925X07000609 18634578

[B33] Hertzler J. I. Rolls M. M. (2024). “Out with the old, in with the new: dendrite degeneration and regeneration,” in Wiring the Nervous System: Mechanisms of Axonal and Dendritic Remodelling in Health and Disease. 1st Edn. Editor Tran T. S. Yaron A. (Abingdon: River Publishers), 107–134. 10.1201/9781032632698-4 38536946

[B34] Hilu-Dadia R. Kurant E. (2020). Glial phagocytosis in developing and mature drosophila CNS: tight regulation for a healthy brain. Curr. Opin. Immunol. 62, 62–68. 10.1016/J.COI.2019.11.010 31862622

[B35] Huang T. T. Matsuyama H. J. Tsukada Y. Singhvi A. Syu R. T. Lu Y. (2020). Age-dependent changes in response property and morphology of a thermosensory neuron and thermotaxis behavior in *Caenorhabditis elegans* . Aging Cell 19, e13146. 10.1111/ACEL.13146 32307902 PMC7253067

[B36] Kulkarni V. A. Firestein B. L. (2012). The dendritic tree and brain disorders. Mol. Cell. Neurosci. 50, 10–20. 10.1016/j.mcn.2012.03.005 22465229

[B37] Lee I. H. Procko C. Lu Y. Shaham S. (2021). Stress-induced neural plasticity mediated by glial GPCR REMO-1 promotes *C. elegans* adaptive behavior. Cell Rep. 34, 108607. 10.1016/J.CELREP.2020.108607 33440160 PMC7845533

[B38] Lewis J. A. Hodgkin J. A. (1977). Specific neuroanatomical changes in chemosensory mutants of the nematode *Caenorhabditis elegans* . J. Comp. Neurol. 172, 489–510. 10.1002/cne.901720306 838889

[B39] Maurya A. K. (2022). Structural diversity in a stereotypic organelle — sensory cilia of *Caenorhabditis elegans* . J. Cell Physiol. 237, 2668–2672. 10.1002/JCP.30732 35686462

[B40] Mehl L. C. Manjally A. V. Bouadi O. Gibson E. M. Tay T. L. (2022). Microglia in brain development and regeneration. Development 149, dev200425. 10.1242/DEV.200425 35502782 PMC9124570

[B41] Ohkura K. Bürglin T. R. (2011). Dye-filling of the amphid sheath glia: implications for the functional relationship between sensory neurons and glia in *Caenorhabditis elegans* . Biochem. Biophys. Res. Commun. 406, 188–193. 10.1016/J.BBRC.2011.02.003 21295547

[B42] Oikonomou G. Perens E. A. Lu Y. Watanabe S. Jorgensen E. M. Shaham S. (2011). Opposing activities of LIT-1/NLK and DAF-6/Patched-Related direct sensory compartment morphogenesis in *C. elegans* . PLoS Biol. 9, e1001121. 10.1371/journal.pbio.1001121 21857800 PMC3153439

[B43] Oikonomou G. Shaham S. (2011). The glia of Caenorhabditis elegans. Glia 59, 1253–1263. 10.1002/GLIA.21084 21732423 PMC3117073

[B44] Perens E. A. Shaham S. (2005). *C. elegans* daf-6 encodes a patched-related protein required for lumen formation. Dev. Cell 8, 893–906. 10.1016/j.devcel.2005.03.009 15935778

[B45] Perkins L. A. Hedgecock E. M. Thomson J. N. Culotti J. G. (1986). Mutant sensory cilia in the nematode *Caenorhabditis elegans* . Dev. Biol. 117, 456–487. 10.1016/0012-1606(86)90314-3 2428682

[B46] Procko C. Lu Y. Shaham S. (2011). Glia delimit shape changes of sensory neuron receptive endings in *C. elegans* . Development 138, 1371–1381. 10.1242/DEV.058305 21350017 PMC3050665

[B47] Procko C. Lu Y. Shaham S. (2012). Sensory organ remodeling in *Caenorhabditis elegans* requires the zinc-finger protein ZTF-16. Genetics 190, 1405–1415. 10.1534/GENETICS.111.137786 22298710 PMC3316652

[B48] Purice M. D. Quitevis J. A. Sean R. Zager M. Setty M. Singhvi A. (2025). Molecular profiling of adult *C. elegans* glia across sexes by single-nuclear RNA-Seq. *Dev. Cell* 0 60, 2659–2678.e10. 10.1016/J.DEVCEL.2025.05.013 PMC12354267 40527319

[B49] Raiders S. Black E. C. Bae A. MacFarlane S. Klein M. Shaham S. (2021). Glia actively sculpt sensory neurons by controlled phagocytosis to tune animal behavior. Elife 10, e63532. 10.7554/ELIFE.63532 33759761 PMC8079151

[B50] Raizen D. M. Zimmerman J. E. Maycock M. H. Ta U. D. You Y. J. Sundaram M. V. (2008). Lethargus is a *Caenorhabditis elegans* sleep-like state. Nature 451, 569–572. 10.1038/nature06535 18185515

[B51] Ray S. Singhvi A. (2021). Charging up the periphery: glial ionic regulation in sensory perception. Front. Cell Dev. Biol. 9, 687732. 10.3389/fcell.2021.687732 34458255 PMC8385785

[B52] Ray S. Gurung P. Manning R. S. Kravchuk A. A. Singhvi A. (2024). Neuron cilia restrain glial KCC-3 to a microdomain to regulate multisensory processing. Cell Rep. 43, 113844. 10.1016/J.CELREP.2024.113844 38421867 PMC11296322

[B53] Razzauti A. Laurent P. (2021). Ectocytosis prevents accumulation of ciliary cargo in *C. elegans* sensory neurons. Elife 10, e67670. 10.7554/eLife.67670 34533135 PMC8492061

[B54] Shaham S. (2005). “Glia–neuron interactions in nervous system function and development,” in Current topics in developmental biology, 39–66. 10.1016/S0070-2153(05)69003-5 16243596

[B55] Shaham S. (2006). Glia-neuron interactions in the nervous system of *Caenorhabditis elegans* . Curr. Opin. Neurobiol. 16, 522–528. 10.1016/J.CONB.2006.08.001 16935487

[B56] Shaham S. (2010). Chemosensory organs as models of neuronal synapses. Nat. Rev. Neurosci. 11, 212–217. 10.1038/NRN2740 20029439 PMC2860653

[B57] Singhvi A. Shaham S. (2019). Glia-neuron interactions in *Caenorhabditis elegans* . Annu. Rev. Neurosci. 42, 149–168. 10.1146/ANNUREV-NEURO-070918-050314 30883261 PMC13730838

[B58] Singhvi A. Liu B. Friedman C. J. Fong J. Lu Y. Huang X.-Y. (2016). A glial K/Cl transporter controls neuronal receptive ending shape by chloride inhibition of an rGC. Cell 165, 936–948. 10.1016/j.cell.2016.03.026 27062922 PMC4860081

[B59] Singhvi A. Shaham S. Rapti G. (2024). Glia development and function in the nematode *Caenorhabditis elegans* . Cold Spring Harb. Perspect. Biol. 16, a041346. 10.1101/CSHPERSPECT.A041346 38565269 PMC11445397

[B60] Stephenson J. R. Purcell R. H. Hall R. A. (2014). The BAI subfamily of adhesion GPCRs: synaptic regulation and beyond. Trends Pharmacol. Sci. 35, 208–215. 10.1016/J.TIPS.2014.02.002 24642458 PMC4029589

[B61] Taylor S. R. Santpere G. Weinreb A. Barrett A. Reilly M. B. Xu C. (2021). Molecular topography of an entire nervous system. Cell 184, 4329–4347.e23. 10.1016/j.cell.2021.06.023 34237253 PMC8710130

[B62] Varandas K. C. Hodges B. M. Lubeck L. Farinas A. Liang Y. Lu Y. (2025). Glia detect and transiently protect against dendrite substructure disruption in *C. elegans* . Nat. Commun. 16, 79–15. 10.1038/s41467-024-55674-0 39747235 PMC11696001

[B63] Wallace S. W. Singhvi A. Liang Y. Lu Y. Shaham S. (2016). PROS-1/Prospero is a major regulator of the glia-specific secretome controlling sensory-neuron shape and function in *C. elegans* . Cell Rep. 15, 550–562. 10.1016/j.celrep.2016.03.051 27068465 PMC4838487

[B64] Wallace S. W. Lizzappi M. C. Magemizoğlu E. Hur H. Liang Y. Shaham S. (2021). Nuclear hormone receptors promote gut and glia detoxifying enzyme induction and protect *C. elegans* from the mold P. brevicompactum. Cell Rep. 37, 110166. 10.1016/J.CELREP.2021.110166 34965433 PMC8733895

[B65] Wang Y. Apicella A. Lee S. K. Ezcurra M. Slone R. D. Goldmit M. (2008). A glial DEG/ENaC channel functions with neuronal channel DEG-1 to mediate specific sensory functions in *C. elegans* . EMBO J. 27, 2388–2399. 10.1038/emboj.2008.161 18701922 PMC2543049

[B66] Wang Y. D’Urso G. Bianchi L. (2012). Knockout of glial channel ACD-1 exacerbates sensory deficits in a *C. elegans* mutant by regulating calcium levels of sensory neurons. J. Neurophysiol. 107, 148–158. 10.1152/JN.00299.2011 21994266 PMC3349695

[B67] Wang L. Graziano B. Encalada N. Fernandez-Abascal J. Kaplan D. H. Bianchi L. (2022). Glial regulators of ions and solutes required for specific chemosensory functions in *Caenorhabditis elegans* . iScience 25, 105684. 10.1016/J.ISCI.2022.105684 36567707 PMC9772852

[B68] Wang J. Barr M. M. Wehman A. M. (2024). Extracellular vesicles. *Genetics* 227. Genetics 227, iyae088. 10.1093/GENETICS/IYAE088 38884207 PMC11304975

[B69] Ward S. Thomson N. White J. G. Brenner S. (1975). Electron microscopical reconstruction of the anterior sensory anatomy of the nematode Caenorhabditis elegans. J. Comp. Neurology 160, 313–337. 10.1002/CNE.901600305 1112927

[B70] White J. G. Southgate J. N. Brenner S. (1986). The structure of the nervous system of the nematode *Caenorhabditis elegans* . Philosophical Trans. R. Soc. Lond. B, Biol. Sci. 314, 1–340. 10.1098/rstb.1986.0056 22462104

[B71] Wilton D. K. Dissing-Olesen L. Stevens B. (2019). Neuron-glia signaling in synapse elimination. Annu. Rev. Neurosci. 42, 107–127. 10.1146/annurev-neuro-070918-050306 31283900

[B72] Wu Q. Cao X. Yan D. Wang D. Aballay A. (2015). Genetic screen reveals link between the maternal effect sterile gene mes-1 and pseudomonas aeruginosa-induced neurodegeneration in *Caenorhabditis elegans* . J. Biol. Chem. 290, 29231–29239. 10.1074/JBC.M115.674259 26475858 PMC4705928

[B73] Wu J. Yarmey V. R. Yang O. J. Soderblom E. J. San-Miguel A. Yan D. (2025). Heat shock proteins function as signaling molecules to mediate neuron–glia communication in *C. elegans* during aging. Nat. Neurosci. 2025, 1635–1648. 10.1038/s41593-025-01989-0 40533573

[B74] Yoshida A. Nakano S. Suzuki T. Ihara K. Higashiyama T. Mori I. (2016). A glial K+/Cl-cotransporter modifies temperature-evoked dynamics in *Caenorhabditis elegans* sensory neurons. Genes Brain Behav. 15, 429–440. 10.1111/gbb.12260 26463820

[B75] Zhang Y. Zhang X. Dai Y. Song M. Zhou Y. Zhou J. (2021). The decrease of intraflagellar transport impairs sensory perception and metabolism in ageing. Nat. Commun. 12, 1789. 10.1038/S41467-021-22065-8 33741976 PMC7979750

